# Peripheral inflammation and neurocognitive functioning in early psychosis: Specific associations of TNF-α and IL-6 with social cognition

**DOI:** 10.1192/j.eurpsy.2025.10063

**Published:** 2025-07-10

**Authors:** Ana Catalán, Claudia Aymerich, José Manuel Rodríguez-Sánchez, Borja Pedruzo, Gonzalo Salazar de Pablo, Patxi Gil, Francisco Aguayo, Garazi Acasuso, Alvaro Collado-Pérez, Javier Goena, Olatz Ibarretxe, Iñaki Zorrilla, Ana González-Pinto, Leire Erkoreka, Daniel Alonso-Alconada, Paolo Fusar-Poli, Miguel Angel González-Torres

**Affiliations:** 1 Department of Neuroscience, University of the Basque Country UPV/EHU, Leioa, Spain; 2 Department of Psychiatry, Basurto University Hospital, OSI Bilbao-Basurto, Bilbao, Spain; 3 Early Psychosis: Interventions and Clinical-Detection (EPIC) Lab, Department of Psychosis Studies, King’s College London, London, UK; 4 Neurosciences, Biobizkaia Health Research Institute, Bizkaia, Spain; 5 Spanish Network for Research in Mental Health, Carlos III Institute (CIBERSAM, ISCIII), Madrid, Spain; 6 Department of Child and Adolescent Psychiatry, Institute of Psychiatry, Psychology & Neuroscience, King’s College London, London, UK; 7 Child and Adolescent Mental Health Services, South London and Maudsley NHS Foundation Trust, London, UK.; 8 Osakidetza, Basque Health Service, Bizkaia Mental Health Service, Lehenak Program, Bilbao, Spain; 9 Department of Child and Adolescent Psychiatry, Institute of Psychiatry and Mental Health, Hospital General Universitario Gregorio Marañón School of Medicine, Universidad Complutense, Madrid, Spain; 10 Department of Laboratory Medicine, Basurto University Hospital, OSI Bilbao-Basurto, Bilbao, Spain; 11 Psychiatry Service, Basque Country Health Service (Osakidetza), Araba University Hospital, Vitoria-Gasteiz, Spain; 12 Neurosciences, BioAraba, Health Research Institute, Vitoria-Gasteiz, Spain; 13 Galdakao-Usansolo University Hospital, Osakidetza Basque Health Service, Galdakao, Spain; 14 Department of Cell Biology and Histology, School of Medicine and Nursing, University of the Basque Country (UPV/EHU), Leioa, Spain; 15 Department of Brain and Behavioral Sciences, University of Pavia, Pavia, Italy; 16 Outreach and Support in South-London (OASIS) service, South London and Maudlsey (SLaM) NHS Foundation Trust, London, UK; 17 Department of Psychiatry and Psychotherapy, University Hospital, Ludwig-Maximilian-University (LMU), Munich, Germany

**Keywords:** clinical high risk, IL-6, psychosis, schizophrenia, TNF-α

## Abstract

**Background:**

Cognitive deficits and immune system dysregulation are core features of psychotic disorders. Among inflammatory markers, interleukin-6 (IL-6) and tumour necrosis factor-alpha (TNF-α) have been linked to both psychosis pathophysiology and related cognitive impairments.

**Methods:**

We investigated associations among IL-6, TNF-α, and neurocognitive performance in 107 participants: individuals at clinical high risk for psychosis (CHR-P, *n* = 35), first-episode psychosis (FEP, *n* = 39), and healthy controls (HC, *n* = 33). Assessments included memory, processing speed, executive function, and social cognition. Cytokines were measured from fasting serum samples. Analyses included ANOVA, correlations, and multivariate regressions controlling for age, sex, IQ, group, and symptom severity.

**Results:**

TNF-α levels were significantly elevated in FEP compared to CHR-P (*p* = 0.0251); IL-6 differences were non-significant. FEP showed poorer performance in multiple cognitive domains, especially social cognition. CHR-P individuals exhibited intermediate profiles between FEP and HC in cognition. In adjusted regression models, IL-6 was significantly associated with undermentalization on the MASC task (*β* = 0.28, *p* = 0.0337) and showed a trend-level association with slower processing speed (*β* = 0.98, *p* = 0.075). TNF-α levels predicted poorer facial emotion recognition (*β* = −1.37, *p* = 0.0022). IQ and group were significant covariates in most models.

**Conclusions:**

Our findings suggest that peripheral inflammation, particularly IL-6 and TNF-α, may selectively impact social cognitive functioning in early psychosis. Though modest, these associations highlight potential inflammatory contributions to functional impairment and support further investigation of immunological targets in early intervention.

## Introduction

Psychotic disorders, including schizophrenia and related conditions, are marked by persistent cognitive impairments [1], neurobiological dysregulation [2, 3], and systemic alterations [4, 5], along with a profound disruption of personal wellbeing [6, 7]. Besides, cognitive dysfunction is a core feature of psychotic disorders [1], often preceding the emergence of positive symptoms and persisting throughout the illness [8–10]. Deficits typically affect attention, working memory, executive functioning, processing speed, and social cognition [8, 11], with significant consequences for functional outcomes and quality of life [12].

A growing body of evidence points to the immune system – and specifically inflammation – as a key factor in the pathophysiology of psychosis [13–15], particularly during its early stages. Among the most studied inflammatory markers are interleukin-6 (IL-6) [16] and tumour necrosis factor-alpha (TNF-α) [17–19], due to their central role in immune activation, neuroinflammation, and potential effects on brain function. IL-6 and TNF-α have been implicated in modulating neurocognitive performance [20], through both direct and indirect pathways, including effects on synaptic plasticity, neurotransmitter systems (e.g., dopamine and glutamate) [21], and oxidative stress [22]. IL-6 is secreted by immune cells, endothelial cells, and adipose tissue in response to infection, injury, and stress [23], while TNF-α is mainly produced by macrophages and monocytes and plays a key role in immune response, apoptosis, and inflammatory signalling [24]. Both cytokines can cross the blood–brain barrier and contribute to neuroinflammation, neuronal signalling, and cognitive impairment [25]. Elevated peripheral levels have been associated with reduced cortical thickness and brain volume in regions relevant to cognition such as attention, visual learning, and verbal fluency [19]. In parallel, negative correlations have been observed between inflammatory markers and cognitive performance in individuals with psychosis [20, 26].

Elevated IL-6 and TNF-α levels have also been consistently observed in individuals at clinical high risk for psychosis (CHR-P) [27] and in first-episode psychosis (FEP) patients [28], suggesting their involvement in illness onset and progression. In CHR-P individuals, higher cytokine levels have also been associated with an increased risk of transition to psychosis [29], supporting their value as potential biomarkers of disease evolution [30]. In patients with FEP of schizophrenia, higher levels of peripheral proinflammatory cytokines were associated with poorer performance in Theory of Mind tasks, highlighting a potential link between immune dysregulation and social cognition deficits early in the illness [16].

In addition to its link with cognitive dysfunction, inflammation has also been associated with the severity of positive symptoms in early psychosis [31, 32]. These associations suggest that immune dysregulation may contribute not only to neurocognitive impairment but also to the clinical expression of psychotic symptoms. Given these findings, IL-6 and TNF-α are increasingly viewed as candidate biomarkers for both psychosis risk and cognitive deterioration. Targeting neuroinflammatory mechanisms may represent a promising avenue for early intervention and preventive strategies and could help deepen our understanding of the potential causal role of cytokines in the pathogenesis of psychosis [33]. However, the relationship between inflammation and neurocognition remains complex, and further research is needed to clarify these associations.

This study aims to investigate the relationship among IL-6, TNF-α, and neurocognitive performance in early psychosis. Specifically, we examine group differences in cytokine levels and cognitive functioning among healthy controls (HC), CHR-P, and FEP participants, and explore the associations between inflammatory markers and cognitive domains within this transdiagnostic sample.

## Materials and methods

### Study design and participants

This study was conducted within the framework of the Prebentziorako Gazte Programa (PREGAP), a longitudinal research initiative investigating individuals at CHR-P and those with FEP (founded by the Department of Health of the Basque Country for research and development projects in health – promotion of health research activity). Participants were recruited from a range of clinical settings, including emergency departments, inpatient units, outpatient clinics, and primary care. Recruitment sites included OSI Bilbao-Basurto (Basurto University Hospital), Lehenak Bilbao (the Bizkaia Mental Health Network), and the Psychiatry Department of Santiago Apostol Hospital in Vitoria-Gasteiz. Baseline assessments and scheduled follow-ups were conducted as part of the study protocol.

### Inclusion and exclusion criteria

CHR-P individuals were identified based on the Comprehensive Assessment of At-Risk Mental States (CAARMS) criteria [34], ensuring that they met established criteria for being at clinical high risk for psychosis. FEP patients met DSM-5-TR criteria for diagnosis, and comorbidities were assessed using the MINI International Neuropsychiatric Interview (MINI) [35]. These patients had experienced psychotic symptoms for less than two years since onset, with diagnoses including schizophrenia, schizoaffective disorder, brief psychotic disorder, psychosis NOS, mood disorders with psychotic features, and substance-induced psychotic disorder. Exclusion criteria included: severe neurological conditions, intellectual disability, and major systemic inflammatory or autoimmune diseases that could confound biomarker analyses. HC were defined as individuals with no current or past psychiatric diagnosis, no first-degree family history of psychotic disorders, and no major neurological, inflammatory, or autoimmune conditions.

### Clinical and cognitive assessments

Each participant underwent a comprehensive assessment covering multiple domains. Clinical data were collected using a comprehensive set of standardized assessments to evaluate symptom severity, functioning, and psychiatric history. Psychotic symptomatology was assessed with the Positive and Negative Syndrome Scale (PANSS) [36] in FEP individuals, while individuals at CHR-P were classified using the CAARMS criteria [34]. Depressive symptoms were measured with the Calgary Depression Scale (CDS) [37], and overall functioning was evaluated using the Social and Occupational Functioning Scale (SOFS) [38], and the Global Assessment of Functioning (GAF) [39]. The Clinical Global Impression (CGI) [40] scale was included to provide a clinician-rated measure of illness severity. Additionally, childhood adversity was assessed using the Childhood Trauma Questionnaire (CTQ) [41].

Cognitive and social cognitive abilities were assessed using a battery of well-established neuropsychological tests. General cognitive functioning was evaluated with selected subscales of the Wechsler Adult Intelligence Scale-IV (WAIS-IV) [42]. Executive functioning and cognitive flexibility were measured using the Wisconsin Card Sorting Test (WCST) [43] and the Stroop Test [44], while processing speed and cognitive flexibility were further assessed with the Trail Making Test A and B (TMT-A, TMT-B) [45]. Verbal fluency was examined through the COWAT task [46], visuospatial memory and organization were assessed using the Rey Complex Figure Test [47], and verbal learning and memory were measured with the Hopkins Verbal Learning Test (HVLT) [48].

Social cognition was evaluated through multiple tasks assessing different domains. Facial emotion recognition was measured using the PERE Facial Recognition Task [49], while the Movie Assessment for Social Cognition (MASC) [50] assessed mentalizing abilities and theory of mind.

### Biomarker collection

A fasting blood sample was collected between 8:00 and 10:00 AM following overnight fasting, in order to minimize potential circadian variation. Samples were used for inflammatory marker analysis, including IL-6 and TNF-α, and broader biometric parameters such as cardiometabolic and hormonal markers (e.g., prolactin, lipid profile, and glucose metabolism). Samples were stored and processed at Laboratory Medicine Department of Basurto University Hospital.

### Statistical analysis

Baseline differences among CHR-P, FEP, and HC were analysed using ANOVA or Kruskal-Wallis tests for continuous variables, and Chi-square tests for categorical variables. Pearson correlation analyses were first conducted to examine the bivariate associations among IL-6, TNF-α, and neurocognitive variables. Given the risk of inflated type I error due to multiple comparisons, these correlations were not interpreted independently. Instead, only cognitive outcomes showing nominally significant associations (*p* < 0.05) with either cytokine were entered into multiple linear regression models. Similar approaches have been employed in previous research investigating inflammation and cognition in psychosis [51].

Linear regression models were used to assess the association between cytokine levels and cognitive performance, adjusting for potential confounders including age, sex, IQ, psychosis risk group, and symptom severity (positive symptom z-score). The models were estimated using ordinary least squares regression, and assumptions (normality, linearity, and homoscedasticity of residuals) were verified. Model fit was evaluated using the *F*-statistic, with significance set at *p* < 0.05.

To minimize the risk of overfitting, we restricted the number of predictors per model and ensured an adequate participant-to-variable ratio, following established guidelines [52]. Only a small set of theoretically relevant covariates was included, and dependent variables were selected based on prior bivariate associations. This strategy was designed to increase model robustness and reduce the likelihood of spurious associations. To control for multiple testing, we applied the Benjamini–Hochberg False Discovery Rate (FDR) [53] correction to the significant associations identified in the regression models.

All analyses were conducted using R software (version 2024.04.1 + 748) [54], and results are reported with standardized coefficients, confidence intervals, and effect sizes where applicable.

### Ethical considerations

The study was approved by the Ethics Committee of the Basque Country, and all participants provided written informed consent prior to enrolment.

## Results

### Socio-demographic and clinical characteristics of the sample

The demographic and clinical characteristics of the sample are summarized in Table 1. The study included 33 HC, 35 CHR-P, and 39 FEP. CHR-P group (mean = 22.4 years; SD = 5.55) was younger than HC (mean = 27.5 years; SD = 3.77) and FEP subjects (27.9 years; SD = 9.27) (*p* < 0.05). IQ scores showed significantly a declining trend across groups, with HC having the highest mean IQ (97.4, SD = 17.3), followed by CHR-P (96.7, SD = 19.8), and FEP presenting the lowest IQ (85.1, SD = 18.6). In terms of sex distribution, no significant differences were found between groups (χ^2^ = 4.01, *p* = 0.13), although descriptively, the FEP group had a higher proportion of male participants (64.1%) compared to CHR-P (42.9%) and HC (45.5%). Regarding ethnicity, most participants in all groups identified as Caucasian, with the highest proportion in HC (93.9%), followed by CHR-P (82.9%) and FEP (71.8%). The proportion of Latin participants increased across groups, from 6.1% in HC to 14.2% in CHR-P and 23% in FEP. Arab and other ethnicities were less represented in the sample.

**Table 1.** Socio-demographic characteristics of the sample

		HC (33)	CHR (35)	FEP (39)
Age (mean, SD)*		27.5 (3.77)	22.4 (5.55)	27.9 (9.27)
IQ (mean, SD)*		97.4 (17.3)	96.7 (19.8)	85.1 (18.6)
IL–6 (mean, SD)		2.56 (0.98)	2.34 (1.29)	3.51 (4.07)
TNF- α (mean, SD)***		7.51 (1.54)	6.62 (1.28)	7.94 (2.64)
Ethnicity (*n*, %)	Caucasian	31 (93.9%)	29 (82.9%)	28 (71.8%)
	Latin	2 (6.1%)	5 (14.2%)	9 (23%)
	Arab	0	1 (2.9%)	1 (2.6%)
	Others	0	0	1 (2.6%)
Employment (*n*, %)	Unemployment	4 (12.12)	7 (20)	7 (17.95)
	Student	9 (27.27)	17 (48.57)	14 (35.90)
	Temporary work disability	0	5 (14.29)	9 (23.08)
	Employed	20 (60.61)	6 (17.14)	9 (23.08)

* Significant difference among HC, FEP, and CHR-P.

** Significant difference among HC, CHR-P, and FEP.

*** Significant difference between FEP and CHR-P.

Regarding employment status, the HC group had the highest proportion of employed individuals (60.61%), compared to 17.14% in CHR and 23.08% in FEP. The CHR-P and FEP groups had a higher proportion of students (48.57 and 35.90%, respectively) compared to HC (27.27%). Notably, temporary work disability was reported exclusively in CHR-P (14.29%) and FEP (23.08%), while no participants in the HC group reported disability.

Regarding IL-6 levels, individuals in the FEP group showed the highest mean concentration (mean = 3.51, SD = 4.07), followed by healthy controls (HC) (mean = 2.56, SD = 0.98) and the CHR-P group (mean = 2.34, SD = 1.29). However, these differences were not statistically significant. For TNF-α, the mean level was highest in the FEP group (mean = 7.94, SD = 2.64), followed by HC (mean = 7.51, SD = 1.54) and CHR-P (mean = 6.62, SD = 1.28). Only the difference between FEP and CHR-P was statistically significant (*p* = 0.0251).

In the FEP group, DSM 5-TR schizophrenia was the most common diagnosis, accounting for 25.6%. Schizoaffective disorder was present in 12.8%, while brief psychotic episodes and psychosis NOS were diagnosed in 10.3%. Bipolar disorder and psychosis induced by substances represented 5.1 and 2.6%, respectively. Delusional disorder and PTSD were less frequent, each comprising 2.6% of the sample.

Within the CHR-P group, most participants, 71.4%, were classified with attenuated psychosis syndrome (APS), followed by BLIPS (brief limited intermittent psychotic symptoms) and APS & BLIPS, which each accounted for 11.4% of cases. A small proportion was categorized as having genetic risk and deterioration (GRD) or GRD & APS, at 2.9% each.

The comorbidity in CHR-P group was as follows: the most prevalent comorbid diagnosis was affective disorders, at 23.8%, followed by anxiety disorders, OCD, depression, and borderline personality disorder were each present in 14.27% of cases. ADHD, adjustment disorder, polysubstance use disorder, ASD, and brief psychotic episodes each accounted for 4.8% of the CHR-P group.

### Neurocognitive characteristics of the sample

Table 2 presents the neurocognitive performance across groups. Overall, individuals with FEP showed the weakest performance in most cognitive domains. Significant differences were found between the FEP group and both CHR-P and HC in verbal fluency (COWAT), visual memory (Rey memory), verbal learning (HVLT), and social cognition (PERE correct total). Processing speed and cognitive flexibility, assessed through TMT A and B, also differed significantly, with FEP participants performing worse than both comparison groups. In the Stroop Word and Stroop Word-Colour conditions, group differences were also significant, with FEP individuals showing greater interference effects. Mentalization abilities measured by the MASC task revealed significantly lower undermentalization scores in the FEP group compared to HC, while the CHR-P group differed from HC in the WCST total score. No significant group differences were found in Rey copy or Stroop Colour.

**Table 2.** Neurocognitive performance characteristics of the sample

	HC (*n* = 32)	CHR-P (*n* = 35)	FEP (*n* = 38)
COWAT	28,3 (10,6)	29,8 (10,5)*	23,6 (10,4)
Rey copy	34,6 (2,61)	34,7 (1,97)	33,8 (2,25)
Rey memory	21,5 (6,95)	23,8 (7,28)*	18,1 (6,60)
TMT A	50,2 (10,1)	52,3 (11,2)	48,7 (9,8)**
TMT B	110,5 (22,3)	115,2 (24,1)	108,3 (20,8)**
Stroop word	80,4 (12,6)	78,5 (14,3)	82,1 (11,9)**
Stroop color^+^	50,3 (9,7)	48,9 (10,5)	52 (9,3)
Stroop word/color***	35,2 (7,1)	34,7 (7,8)	36,5 (6,9)
HVLT total	18,7 (5,2)	19,4 (5,8)	17,9 (5,8)**
PERE correct total	14,2 (3,9)	13,8 (4,1)	14,5 (3,7)**
	HC (*n* = 24)	CHR-P (*n* = 30)	FEP (*n* = 30)
MASC correct	10,3 (2,4)^+^	9,7 (2,8)	10,8 (2,2)
MASC overmentalization	5,6 (1,9)	6,1 (2)	5,3 (1,8)
MASC undermentalization	4,2 (1,7)^+^	4,8 (1,9)	3,9 (1,5)
MASC no mentalization	7,5 (2,3)	8,1 (2,6)	7,2 (2,1)**
	HC (*n* = 24)	CHR-P (*n* = 26)	FEP (*n* = 32)
WCST total	23,4 (6,8)^++^	22,5 (7,2)	24,1 (6,5)

* Significant difference between CHR-P and FEP.

** Significant difference between FEP and CHR-P; and significant difference between FEP and HC.

*** Significant difference between all groups.

+ Significant differences between HC and the other groups.

++ Significant differences between HC and CHR-P.

### Relationship between neurocognition and IL 6 and TNF- 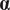


**Figure 1.**
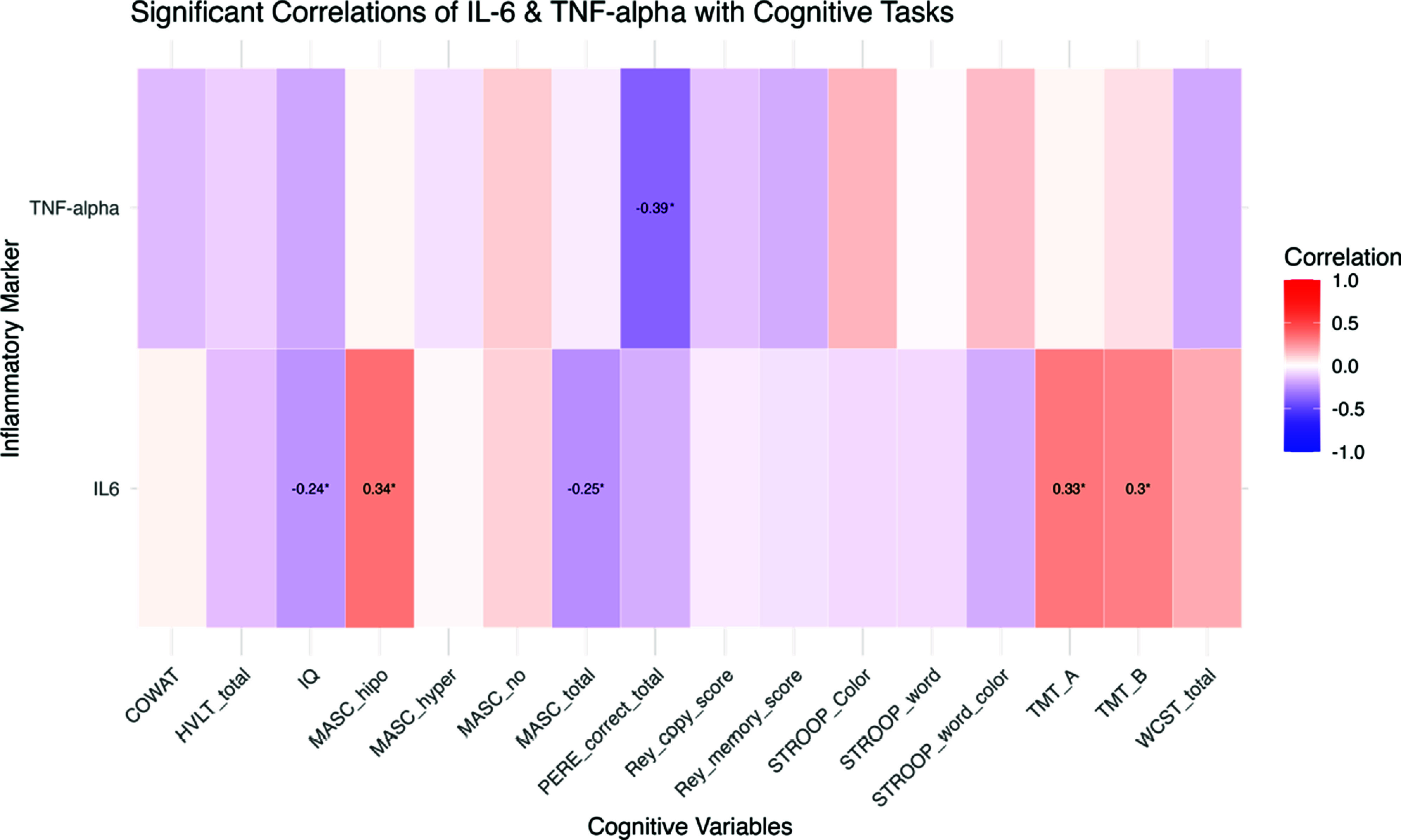
Correlation matrix of inflammatory markers (IL-6 & TNF-α) and cognitive tasks. COWAT, Controlled Oral Word Association Test; HVLT, Hopkins Verbal Learning Test; MASC, Movie for the Assessment of Social Cognition; PERE, Emotion Recognition Task; TMT, Trail Making Test; WCST, Wisconsin Card Sorting Test.

The heatmap (Figure 1) illustrates the strength and direction of the bivariate correlations, with red tones indicating positive correlations and blue tones representing negative correlations. Overall, IL-6 exhibited weak to moderate associations with some of the cognitive performance tests. IL-6 showed positive correlations with TMT A (*r* = 0.33, *p* = 0.0018) and TMT B (*r* = 0.30, *p* = 0.0049), although these associations were of small magnitude. Additionally, a negative correlation was observed between IL-6 and MASC total (*r* = −0.25, *p* = 0.0389) and IQ (*r* = −024, *p* = 0.0257), while a positive correlation was found between IL-6 and MASC undermentalization (*r* = 0.34, *p* = 0.0041). Regarding TNF-α, a stronger negative correlation was found with PERE test (*r* = −0.39, *p* = 0.0002).

Multiple linear regression models were conducted to examine the relationship among IL-6, TNF-α levels and cognitive performance while adjusting for IQ, sex, age, psychosis risk group (HC, CHR, and FEP), and positive symptom severity. We introduced into the model as dependent variables those with positive associations in the bivariate correlations.

IL-6 exhibited no association with TMT-A (*p* = 0.075). The FEP group demonstrated a significant positive association with TMT-A (*β* = 12.23, *p* = 0.0013), indicating that individuals in this group had significantly longer completion times compared to the reference group. Neither sex nor positive symptoms were significantly associated with TMT-A completion time. The model accounted for 24% of the variance in TMT-A (*R*^2^ adjusted = 0.24).

The association between TMT-B performance and IL-6 levels was significant overall (*F* (7, 75) = 9.52, *p* < 0.001), explaining approximately 47% of the variance in TMT-B scores (adjusted *R*^2^ = 0.42). Higher IL-6 levels were not significantly associated with TMT-B performance (*p* = 0 .19). In contrast, lower IQ (*p* < 0.001), older age (*p* = 0.038), and FEP group (*p* = 0.001) were significant predictors of poorer performance. Sex and positive symptoms did not show significant effects.

For MASC Total (*F* (7, 57) = 6.84, *p* < .001), IL-6 was not significantly associated with overall mentalizing performance (*β* = −0.25, *p* > 0.05). However, IQ was positively associated with mentalizing performance (*β* = 0.069, *p* = 0.015), indicating that higher IQ scores relate to better mentalizing ability. HC outperformed other groups in mentalizing accuracy (*β* = 5.71, *p* = 0.0018, *R*^2^ adjusted = 0.40).

In the case of MASC undermentalization (*F* (7, 57) = 4.12, *p* = 0.001), IL-6 showed a significant positive association with undermentalization (*β* = 0.28, *p* = 0.0337), suggesting that higher IL-6 levels may be linked to a tendency to under-attribute mental states to others. IQ showed a trend toward significance (*β* = −0.034, *p* = 0.054), implying that lower IQ may be related to increased undermentalization. HC exhibited lower undermentalization scores compared to other groups (*β* = −2.48, *p* = 0.031). The model explained 25% of the variance (*R*^2^ adjusted = 0.25).

Regarding the PERE recognition test (*F* (6, 77) = 3.60, *p* = 0.003), TNFα showed a significant negative association with performance (*β* = −1.37, *p* = 0.0022), suggesting that higher TNFα levels may be linked to poorer facial emotion recognition. No other variables showed significant effects in this model. The model explained 16% of the variance (*R*^2^ = 0.16).

After applying the Benjamini–Hochberg FDR correction for multiple comparisons to the two significant associations identified in the regression models. Both the association between TNF-α and emotion recognition (PERE) (adjusted *p* = 0.0044) and the association between IL-6 and undermentalization (MASC) (adjusted *p* = 0.0337) remained statistically significant after correction.

## Discussion

In this study, we examined the associations between inflammatory markers and neurocognitive performance across individuals at CHR-P, patients with FEP, and HC. As expected, the FEP group exhibited the most pronounced cognitive impairments, particularly in verbal fluency, memory, processing speed, and social cognition. Adjusted analyses revealed two significant associations between inflammation and social cognition. Higher IL-6 levels were independently associated with increased undermentalization on the MASC task, suggesting a link between systemic inflammation and difficulties in inferring others’ mental states. Additionally, elevated TNF-α levels were negatively associated with performance on the PERE test, indicating a potential impact on facial emotion recognition. These findings remained significant after accounting for key confounders, highlighting that specific aspects of social cognition may be particularly sensitive to peripheral immune dysregulation in early psychosis.

Given prior evidence linking inflammatory markers with the severity of positive symptoms in early psychosis [32], we included positive symptom severity as a covariate in our models. Although our study was not primarily designed to test this association, exploratory analyses in the FEP group supported its relevance, and future studies should consider this dimension when examining immuno-cognitive interactions.

The neurocognitive profile observed in our sample reinforces the notion that cognitive impairments are a core feature of psychotic disorders [1], with the FEP group showing the most pronounced deficits across domains such as verbal fluency, memory, processing speed, and cognitive flexibility [55]. These findings are consistent with previous literature indicating that such deficits are already present at illness onset and tend to persist over time [56]. The intermediate performance of the CHR-P group suggests that subtle cognitive alterations may emerge even before the onset of frank psychosis [8]. In mentalization abilities, significant group differences were observed in the MASC task: the HC group demonstrated better accuracy in identifying mental states, while FEP individuals showed a greater tendency toward undermentalization [57], a pattern that has been linked to social cognition deficits in schizophrenia. The absence of significant differences in tasks such as Rey copy and Stroop Colour further highlights the domain-specific nature of these alterations, underlining the importance of targeted cognitive and social-cognitive assessment in early detection strategies.

Our finding of a robust association between higher TNF-α levels and IL-6 and poorer performance in facial emotion recognition and undermentalization aligns with experimental evidence suggesting a direct impact of systemic inflammation on social cognitive processes. For instance, experimental studies show that mild immune activation, such as with typhoid vaccine or IF-α, can impair social cognition and induce negative emotion processing biases, even in the absence of mood changes [58, 59]. While our study used a cross-sectional design, the results are consistent with the idea that inflammation can selectively impair the neural mechanisms involved in decoding emotional expressions, particularly in vulnerable clinical populations. The robustness of this association, in contrast to the nonsignificant findings for broader cognitive tasks, may reflect a particular vulnerability of socio-affective processing systems to inflammatory dysregulation in early psychosis.

One possible explanation for these specific associations with social cognition, is that complex cognitive functions – such as mentalization and emotion recognition – rely on distributed neural networks that are especially vulnerable to inflammatory disruption. Proinflammatory cytokines like IL-6 and TNF-α can impair synaptic plasticity, reduce prefrontal connectivity, and disrupt neurotransmitter regulation [60-63], all of which are essential for social cognitive processing. Experimental findings have shown that inflammatory mediators (e.g., COX-2) can inhibit synaptic strength and modulate performance in cognitive tasks, particularly in the prefrontal cortex [64].

Moreover, glial cells – particularly microglia – play a critical role in mediating the effects of inflammation on the brain [65, 66]. Upon activation, microglia release proinflammatory cytokines, which can initiate and sustain neuroinflammatory cascades. These cytokines have been shown to induce oxidative stress, alter synaptic architecture, and compromise blood–brain barrier integrity, all of which may disrupt the finely tuned networks underlying social cognitive processes [67]. Cytokine release and glial activation may jointly drive neuronal dysfunction in psychiatric and neurodegenerative disorders, underscoring their relevance in early psychosis [68].

TNF-α and IL-6 showed specific associations with emotion recognition and undermentalization, respectively, suggesting that proinflammatory cytokines may selectively disrupt prefrontal–limbic circuits underlying social cognition in early psychosis [69–71].

### Strengths and limitations

This study presents several strengths. It includes a well-characterized clinical sample covering the psychosis spectrum, with both CHR-P and FEP participants, enabling meaningful comparisons across illness stages. A key procedural strength of this study is the standardized timing of blood collection, which was conducted between 8:00 and 10:00 AM after overnight fasting. This protocol aimed to minimize circadian variation in cytokine levels, particularly for IL-6 [72] and TNF-α, which are known to follow diurnal secretion patterns. Controlling for timing reduces potential measurement noise and improves the comparability of inflammatory marker data across participants. The use of a comprehensive cognitive battery, alongside the assessment of inflammatory markers, allowed for an integrative analysis of neuroimmune-cognitive interactions. Importantly, multivariate models were employed to control for key confounding variables, including IQ, age, sex, and symptom severity, enhancing the reliability of the observed associations.

Nonetheless, some limitations should be considered. The cross-sectional nature of the study prevents conclusions regarding causality or the temporal relationship between inflammation and cognitive functioning. Cytokine levels were measured at a single time point, limiting the ability to capture fluctuations in inflammatory status. The relatively small sample size, particularly in subgroup analyses and regression models with multiple covariates, may have reduced statistical power to detect subtle effects. In addition, missing data in some variables led to reduced effective sample sizes, which further constrained analytical precision. While the cognitive battery covered key domains, certain aspects of social cognition, such as theory of mind and attributional style, were not extensively explored. Finally, the use of bivariate correlations and stepwise regression models may have increased the risk of overfitting and biased parameter estimation. Although efforts were made to reduce this risk by limiting predictors and adjusting for key covariates, more robust statistical approaches (e.g., penalized regression or cross-validation) may strengthen future analyses.

## Conclusion

Our findings suggest that TNF-α and IL-6 are selectively associated with impairments in social cognition – specifically emotion recognition and undermentalization – in early psychosis, highlighting their potential as biomarkers and therapeutic targets. Clinically, incorporating inflammatory markers into early assessments may help identify patients who could benefit from tailored interventions aimed at improving social functioning.

## Data Availability

The data underlying this article are not publicly available due to the confidential nature of the clinical information. These data can, however, be made available from the corresponding author upon reasonable request, subject to approval by the relevant ethics committee and data-sharing agreements.
